# Validation of the Hungarian version of the 6-item turnover intention scale among elderly care workers

**DOI:** 10.1038/s41598-024-66671-0

**Published:** 2024-07-06

**Authors:** Zsanett Németh, Petra Deák, Réka Szűcs, Alexandra Makai, Márta Hock

**Affiliations:** 1 Doctoral School of Health Sciences, University of Pécs, Pécs, 7621 Hungary; 2 Toldy Ferenc Hospital and Clinic, Cegléd, 2700 Hungary; 3 Institute of Physiotherapy and Sport Sciences, Faculty of Health Sciences, University of Pécs, Pécs, 7621 Hungary

**Keywords:** TIS-6, Validation, Turnover intention, Workplace stress, Burnout, Elderly care, Human behaviour, Risk factors

## Abstract

This research examines the psychometric characteristics and reliability of the 6-item turnover intention scale (TIS-6) by Bothma and Roodt (SA J Hum Resour Manag 11:a507, 2013) on a Hungarian sample. The internal validity of the TIS-6 was assessed using data from 269 Hungarian elderly care institution workers. Confirmatory factor analysis was performed to analyse the structural validity. Convergent and discriminant validity were examined with questions on job characteristics and using the Maslach Burnout Inventory and Effort-Reward Imbalance Scale. IBM SPSS 28.0 software was used for the statistical analysis, and the results were considered significant at p < 0.05. The internal consistency of the questionnaire's scale proved to be acceptable (α = 0.826). Convergent validity was confirmed by the relationships between the components of the questionnaire and burnout (r_s_ = 0.512; p < 0.001; r_s_ = 0.419; p < 0.001) and workplace stress (r_s_ = 0.565; p < 0.001; r_s_ = 0.310; p < 0.001). There were significant differences between the TIS-6 scores among the groups with different degrees of burnout (p < 0.001), which indicated adequate discriminant validity of the questionnaire. The structural validity of the questionnaire was acceptable, and the scale questions fit well. The Hungarian version of the TIS-6 scale is a valid and reliable tool for assessing turnover intention among elderly care institution workers in Hungary.

## Introduction

Nursing shortages are a critical issue for healthcare services both locally and globally^1–3^. The World Health Organization (WHO) reported that Central European countries should address nursing shortages^4^. In Hungary, there were 33.33 healthcare and social workers per thousand people in 2021^5^. Turnover intention and the intention to leave the nursing career and migrate abroad are widespread problems in Hungary^6^. The number of people that choose the nursing profession at Hungarian universities is constantly decreasing; therefore, to find a balance and sustainable migration, the right to migrate among nurses must be considered^7^.

Hungary has a high employee turnover rate, with 23% of workers quitting their jobs within a year on average. Over 10% of elderly care is lacking in primary care, and 65% is inadequate in specialized elderly care^8^. The number of published data regarding turnover rates and costs for nurses and elderly care workers in Hungary is quite low^9^. Intentions to leave the nursing profession influence the institution's expenses in Europe by 5–17%^10^.

Many countries were struggling with health workforce shortages even before the COVID-19 pandemic and it created additional challenges in terms of retaining the remaining workforce^11^. The social and psychological effects of the pandemic increased the mean nurses’ turnover intention rate^12^. Before the pandemic, the nurses’ turnover was most often caused by job satisfaction^13^, management and leader style^14–16^, supervisory support^15,16^, supportive work environment^14,16,17^, salary^17^, and work-family balance^18^. During the COVID-19 pandemic, the fear of COVID-19 exposure, stress, and anxiety mostly influenced nurses' intention to change jobs^11,12^.

The solution to the problem of employee turnover has received increasing attention, not only among nurses and in healthcare institutions but also in elderly care and nursing homes^19–21^. To retain nurses, it is necessary to reduce their turnover intention^22^. Identifying the antecedents of turnover intention is crucial since it has been widely researched and proven that turnover intention is an important predictor of actual turnover behaviour^23,24^.

Job stress and burnout have direct and significant effects on the intention to change jobs, but employees who turnover have greater burnout^25,26^. Job satisfaction is believed to be an essential factor for turnover intention^13^, as job satisfaction influences the level of stress experienced at work and burnout^27^. The more committed the employee is, the fewer negative effects the worker experiences physically and mentally during the work, the greater the productivity will be, and the lower the employee’s chances of turnover or absence will be^27^. Job satisfaction has been the subject of numerous studies to identify the causes of employee turnover. Those who are more satisfied with their work have a lower intention to leave the job, but greater job stress, workload or conflict with colleagues that leads to greater turnover intention^25,28^. Effort reward imbalance has a direct effect on burnout and turnover intentions, these have some indirect effect on these outcomes through job satisfaction^29^.

Based on previous studies, the TIS-6 was created with the aim of using a statistically validated measuring tool to measure the prediction of employees’ intention to leave the workplace^30^. Its validity has already been proven in many countries, in different languages and sociocultural environments (e.g., China, Pakistan, India). It has good psychometric characteristics, an adequate level of reliability and discriminant and nomological validity^31–33^.

To the best of our knowledge, there is no internationally developed and acknowledged tool for measuring job turnover that has been validated in the Hungarian language. The aim of this study is to adapt the TIS-6^30^, which is the most internationally cited job turnover questionnaire, to a Hungarian sample and to test its validity and reliability.

## Materials and methods

### Sample and settings

This study used a quantitative research method. The data of the cross-sectional study were collected between June 2022 and May 2023. We contacted the managers of the elderly care institutions in Hungary by email, informed them about the research, invited their employees to fill the questionnaire. We also shared the questionnaire with professional groups on Facebook and asked the appropriate members to complete it. The research participants were asked to complete a structured electronic survey using Google Forms without assistance. The participants were also informed about the study, and their online consent was obtained. The participants in this study were frontline workers or managers/leaders of social elderly care institutions throughout Hungary. The completion of the online questionnaire was voluntary and anonymous. The inclusion criteria were they had to be working in a social elderly care institution in Hungary and had to be at least 18 years of age. Those who were unemployed, nonprofessional staff (e.g., maintenance workers), had a passive employee status (e.g., pregnancy) or working from home were excluded from the study. A total of 269 workers (male = 27; female = 242) from various Hungarian elderly care institutions completed the questionnaire.

### Measurements

The study used the following tools:

#### Demographic questionnaire

The data was collected via a demographic questionnaire, which was prepared by the research team. The demographic section included questions regarding gender, age, education, job position and monthly income of the participants.

#### Turnover intention scale (TIS-6)

The turnover intention scale (TIS-6) was used to collect the data to evaluate turnover intention. The TIS-6 consists of 1, 3, 4, 6, 7, and 8 items from the original TIS-15. The six-item version of the scale was developed by Roodt and Bothma^30^. Each item is assessed on a five-point Likert scale ranging from disagree (1 point) to agree (5 points), each item measures turnover intention, there are no items measuring incongruence. The midpoint of the scale is 18. A total score below 18 indicates the intention to stay, while a total score above 18 indicates the intention to leave. The Cronbach’s alpha of the scale was 0.80^30^. The TIS-6 has excellent psychometric characteristics^30^. The authors of the questionnaire have given us the permission to use the questionnaire for scientific research purposes. The translation into Hungarian was based on international practice.

#### Effort-reward imbalance scale (ERI)

To measure workplace stress, the validated version of the Effort-Reward Imbalance (ERI) questionnaire was used^34^. According to the original version of the scale, if the effort at work is high while the level of the received reward is inadequate, the resulting internal dissatisfaction and stress will increase the risk of stress-related disorder incidents and physical and mental illnesses^35^. ERI can affect turnover intention through direct mediators of depressive symptoms^36^. The validated and shortened version of the Hungarian questionnaire consists of 15 items and three main dimensions: workplace efforts (3 items), rewards (6 items) and overcommitments (6 items)^34,37^. The questions are rated on Likert scale. The score on the Effort Scale ranges from 3 to 15, and the score on the Rewards Scale from 6 to 30.

For the effort scale, the greater the score, the greater the perceived effort, a lower score indicates less perceived reward. There are no questions in this scale to detect incongruent answers.

#### Maslach Burnout Inventory (MBI)

The original questionnaire assessed the frequency and intensity of perceived burnout among individuals in helping professions. The questionnaire contains 22 questions and comprises of three dimensions: emotional exhaustion (EE), depersonalization (Dep) and personal accomplishment (PA).The questionnaire does not contain an L scale or questions to detect incongruent answers. The participants were evaluated on a 7-point Likert scale. Burnout is indicated by high cumulative scores of emotional exhaustion and depersonalization^38^. The questions of the personal effectiveness dimension are to be evaluated in reverse^39^.

### Research procedure

#### Translation of TIS-6

The authors of the original scale were contacted and asked for permission to adapt the scale to the Hungarian language version and to test its validity on a Hungarian sample. The forward-backwards translation method was applied to adopt the TIS-6 to Hungarian culture. The translation was performed based on Beaton’s guideline^40^.

Based on the adaptation process between cultures, in the first phase, two instruments were made by a linguist and a psychologist from the original language (English) to the target language (Hungarian). The translated questionnaire did not contain special medical or academic terms. After that, the two translations were synthesized, and a common translation was made from the first and the second translation. In the third phase, the other linguist and an English teacher blindly translates the Hungarian version of the questionnaire back to English, without prior knowledge of the original questionnaire. To finalize it, a committee consisting of experts (health and social professionals) and translators helped to compare the versions of the questionnaire and to develop the final version of the questionnaire, in preparation for field testing.

In addition to the cultural suitability, the translation was assessed separately by six people to evaluate its comprehensibility. There was no marked difference in the comprehensibility or wording of the questions between the six evaluated persons. In the end, all six questions remained.

#### Test–retest reliability

The next phase was the pre-test. A pilot study was conducted on 22 individuals to assess the comprehensibility and clarity of the finalized and approved Hungarian translation. The Cronbach’s alpha coefficient was determined to be 0.835 for the pre-test. The measurement was repeated with the same participants after one month (Cronbach’s alpha = 0.814). The participants in the pilot test and the study sample were different people. No obvious problems were found during the pilot test, and no further changes were necessary.

### Statistical analyses

The IBM SPSS 28.0 program package was used to prepare the analyses. Descriptive statistics were used to report the sample characteristics. The reliability of the scales was examined by calculating the Cronbach's alpha coefficient (α), which reflects the internal consistency of the items. The corrected item-total correlation coefficient was used to represent the strength of the relationship between each item and the total score of the scale. A value greater than 0.50 means that the items are highly correlated, while scores between 0.30 and 0.50 are considered acceptable^41^. The Shapiro‒Wilk normality test was used to check whether a continuous variable followed a normal distribution^42^. The test-retest reliability of the TIS-6 scale was measured by the Wilcoxon test and interclass correlation coefficients (ICCs with 95% confidence intervals). Convergent validity was assessed by Spearman’s rank correlation coefficients on the TIS-6, MBI and ERI results to compare the factor structure of the measuring instruments. To examine the extent of the difference between the constructions, discriminant validity tests were performed using Mann‒Whitney *U* tests. Confirmatory factor analysis (CFA) was performed to examine structural validity, and the proposed measurement theory was applied by determining the factor loadings of the items, as well as χ^2^, degree of freedom (df), χ^2^/df, goodness-of-fit index (GFI), Tucker‒Lewis index (TLI), degree of fit of comparative fit index (CFI) and root mean square error of approximation (RMSEA) indices. The loading values of the factor analysis describes the relationships between the factors and the observed variables, where factor loadings between 0.30 and 0.59 are considered moderate, and values above 0.60 are considered high^43^. Cronbach's alpha values closer to one indicate higher internal consistency, while values closer to zero indicate lower internal consistency, and values above 0.70 are considered reliable^44^. A χ^2^/df ratio less than 5 indicates an acceptable fit between the assumed model and the sample data^45^. For the RMSEA, values below 0.05 indicate a good fit, while values below 0.08 indicate an acceptable fit; however, indices above 0.10 are unacceptable^46^. The GFI, TLI and CFI are between 0 and 1, where values above 0.95 indicate a good fit, and values above 0.90 indicate an acceptable fit^47^. The aim was to test the replicability of the original factor structure of the scale on the Hungarian sample using IBM AMOS 29.0 software^48^. The final Hungarian version of the TIS-6 can be found in Supplementary Table S1 online.

### Ethical considerations

Ethical approval for the study was granted by the Hungarian Medical Research Council Scientific and Research Ethics Committee (BMEÜ/599-3/2022/EKU). All procedures performed in this study were in accordance with ethical standards and with the 1964. Helsinki declaration. The protocol was registered on clinicaltrials.gov with the identifier NCT05487157

## Results

### Descriptive statistics

The present study sample consisted of elderly care institution workers in Hungary. A completed questionnaire was returned by 277 participants. After filtering out questionnaires that were incorrectly completed and those filled by workers who did not meet the inclusion criteria (8 questionnaires); we ultimately obtained a total of 269 completed questionnaires. The mean age of the participants was 46.72 (SD = 8.91) years, and 90.0% were female. The descriptive statistics of the variables are presented in Table 1. There was no significant difference (p > 0.05) between the groups shown in Table 1, and the TIS-6 total score.

**Table 1:** Demographic and occupational characteristics of the participants (N = 269).

Variables	N	%
Age group		
35 years old or younger	31	11.52
30–50 years old	144	53.53
50 years old or older	94	34.94
Educational attainment		
Below high school	62	23.05
High school with graduation	86	31.97
Bachelor’s degree or above	121	44.98
Job position		
Frontline nurses (social workers, nurses)	138	51.30
Workers with management role(s) (head of nurse/institution, leader, administrator)	84	31.22
Therapists, mental health workers	47	17.47
Place of work		
Village	105	39.03
City	98	36.43
County seat	53	19.70
Capital city	13	4.83
Working experience		
< 3 years	29	10.78
3–10 years	67	24.90
11–20 years	86	31.97
21 years or above	87	32.34
Monthly income HUF (1 US$ is approximately 340.-HUF)		
200.000.-HUF or below	25	9.29
200.001–400.000.-HUF	188	69.88
400.000.-HUF or above	56	20.81

### Internal consistency and test–retest reliability

Mean TIS-6 score was 14.75 (SD = 5.54). The internal consistency of the TIS-6 scale was 0.826, indicating very good internal reliability. The corrected total item correlation values were between 0.334 and 0.742, and the skewness and kurtosis values suggested that items 1, 3, 4, and 5 were not normally distributed; however, all the items were found to be reliable (Table 2). The Shapiro‒Wilk normality test confirmed that the data was not normally distributed (p < 0.01). The test–retest reliability of the scale was estimated by performing the same test with 22 participants who were assessed twice over a 4-week period. The Cronbach’s alpha of the test results was 0.835, while that of the retest results was 0.814, so they were reliable. Based on the Pearson correlation, the test–retest total results showed that the first and second measurement results were similar (r = 0.791, p < 0.001). The Wilcoxon signed rank test was used to evaluate the difference between the means of the total test-retest points, and no statistically significant difference was found between the two measurements (p = 0.365; t = − 0.905). The test–retest reliability of the TIS-6 was acceptable (ICC (95% CI) = 0.884 (0.720–0.952)).

**Table 2:** Descriptive details for the Hungarian TIS-6 (N = 269).

Item	Mean ± (SD)	Mean std. error	Skewness	Kurtosis	Ranges	Corrected item total correlation	Cronbach’s α when deleted
1	2.11 ± (1.200)	0.73	0.822	− 0.260	4	0.742	0.768
2	2.77 ± (1.152)	0.70	0.106	− 0.626	4	0.334	0.846
3	2.25 ± (1.189)	0.72	0.574	− 0.577	4	0.696	0.778
4	2.41 ± (1.389)	0.85	0.569	− 0.970	4	0.680	0.779
5	2.42 ± (1.498)	0.91	0.551	− 1.159	4	0.621	0.795
6	2.80 ± (1.099)	0.67	0.090	− 0.452	4	0.528	0.812

Overall Cronbach’s α = 0.826.

### Convergent and discriminant validity

Convergent validity was supported by the significant correlations with turnover and burnout and workplace stress (N = 269). In our sample, the Cronbach's alpha of the subscales of the ERI scale was 0.665 for the effort subscale, 0.806 for the reward subscale, and 0.675 for overcommitment. The Cronbach’s alpha values of the subscales of the MBI are EE = 0.869, Dep = 0.700, and PA = 0.841. The turnover intention was significantly and positively correlated with the results of two dimensions of the MBI questionnaire, EE (r_s_ = 0.512; p < 0.001) and Dep (r_s_ = 0.419; p < 0.001), as well as with the effort (r_s_ = 0.565; p < 0.001) and imbalance (r_s_ = 0.310; p < 0.001) dimensions of the ERI questionnaire. The average TIS-6 score was 17.80 (SD = 5.65) points for those with a high degree of burnout (N = 69) and 13.71 (SD = 5.11) points for workers with a low or moderate degree of burnout (N = 200). There was a significant difference between the two groups (p < 0.001).

### Confirmatory factor analysis (CFA)

**Figure 1:**
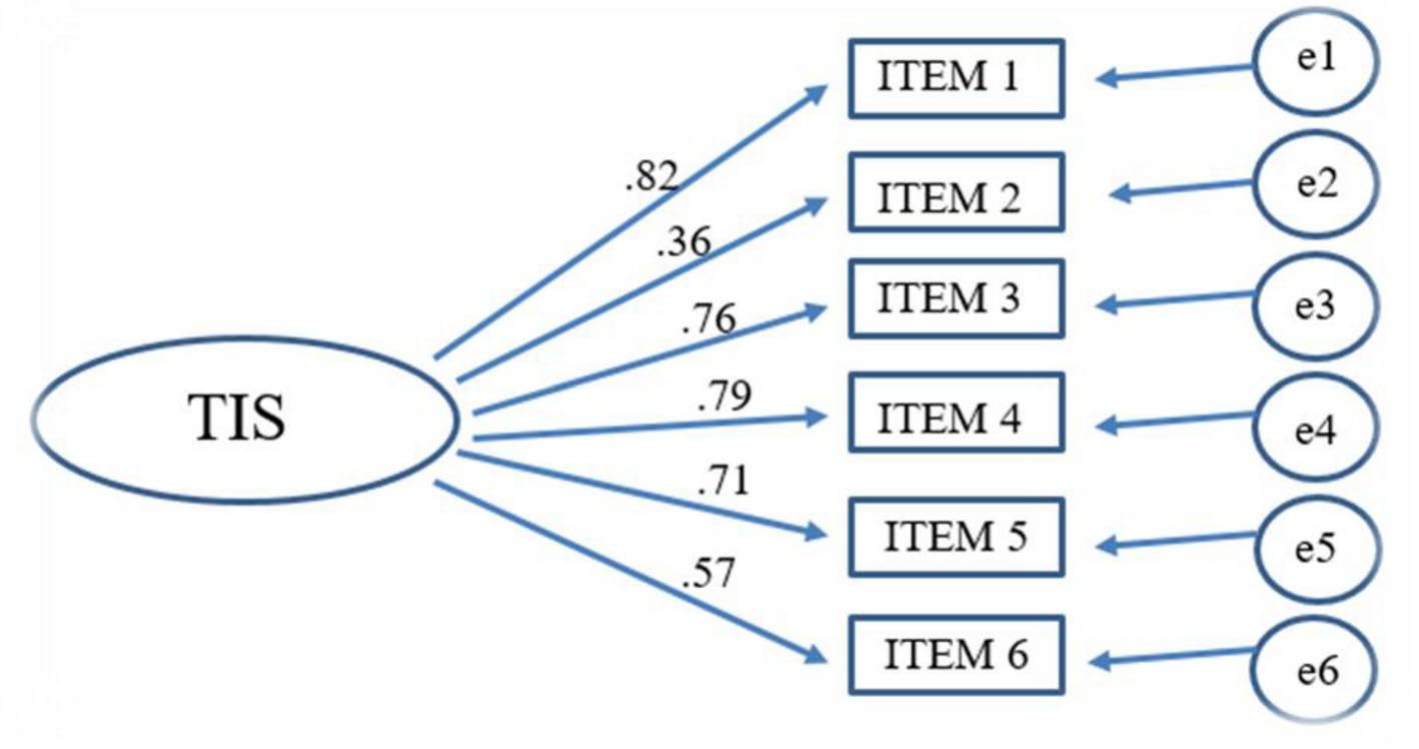
The results of the final CFA model of the Hungarian TIS-6. Note: TIS = 6-item turnover intention scale, ITEM 1 = TIS-1 item, ITEM 2 = TIS-2 item, ITEM 3 = TIS-3 item, ITEM 4 = TIS-4 item, ITEM 5 = TIS-5 item, ITEM 6 = TIS-6 item, e1 = Error variance for ITEM 1, e2 = Error variance for ITEM 2, e3 = Error variance for ITEM 3, e4 = Error variance for ITEM 4, e5 = Error variance for ITEM 5, e6 = Error variance for ITEM 6.

The unifactorial factor structure was tested to examine the structural validity of the TIS-6 questionnaire. The sample size was calculated based on the previous published articles, a ratio of ten participants per item for factor analysis^49^. During the structural validity test, all fit indices were acceptable, except for the results of the χ^2^ test; detailed values are provided in Table 3. The factor loadings of the items were tested via confirmatory factor analysis (CFA), and the results shown in Fig 1 reported that the scale measured turnover intention well and that the construct validity was adequate.

**Table 3:** Indices for CFA of TIS-6 (N = 269).

Model fit	Degree of freedom (df)	Chi-square (χ^2^)	χ^2^/df	p	Comparative Fit Index (CFI) (≤ 0.950)	Tucker‒Lewis Index (TLI) (≤ 0.950)	Root Mean Square Error (RMSEA) (≤ 0.050)
Factor-1	9	20.688	2.299	0.014*	0.980	0.952	0.069

*p < 0.05.

## Discussion

This study reported the validity and reliability of the Hungarian version of the TIS-6 by examining its psychometric characteristics. The psychometric properties of the TIS-6 questionnaire have been studied in several international studies, and it has already been tested on Chinese, Pakistani and Indian samples^31–33^. The validity of the TIS-6 was determined using linguistic, content and construct validity methods.

In this study, an intermittent method was chosen in test-retest method. The reliability of the scale was checked with the same test administered twice with the same 22 participants over a 4-week period. The test–retest results proved to be reliable based on the Cronbach’s alpha values of the test results. To measure the linear correlation between the two sets of data, the Pearson correlation test was used. The first and second measurements were similar, and there were no statistically significant differences between the two measurements. The Wilcoxon signed rank test was used to check whether the two dependent samples differed significantly from each other, and no statistically significant difference was found between the two measurements, thus confirming that the first and second measurements were similar. These findings show that the test–retest reliability of the TIS-6 was acceptable.

Based on the international validation results, the internal consistency of the TIS-6 varied between 0.700 and 0.870, which also indicate good reliability^30,31,33^. The Cronbach’s alpha coefficients of the Hungarian TIS-6 showed that the content validity of the scale was very good, with a value of 0.826. The Cronbach's alpha total value was acceptable, so the original set of questions was retained^50^. Based on these results, the internal consistency of the scale can be considered to be high, and we can conclude that the scale items were easy to understand. We also checked the convergent validity of the TIS-6 with other scales (MBI, ERI) and found a significant relationship. Our convergent and discriminant validity results were supported by significant positive correlations with turnover intention, burnout and workplace stress (p < 0.001), which was also confirmed by the results of recent years. Depressive symptoms and effort-reward imbalance act as direct mediators causing intentions to change jobs^36,51^. An imbalance between the level of effort expended and the reward received leads to turnover intention, and exhaustion is the link between ERI and turnover intention^1,52^.

The factor loadings of our scale items ranged between 0.36 and 0.82, so the scale had an acceptable factor loading and confirmed that the scale measured turnover intention well and that the construct validity was adequate. In previous TIS-6 validation studies, factor loadings were also moderately to highly acceptable, ranging between 0.46 and 0.78^31,33^. In our study, we calculated the χ^2^/df value to be 2.229, which indicates an acceptable fit. Based on previous studies, the questionnaire showed a satisfactory fit, with χ^2^/df ratios of 1.17 and 1.24^31,33^. The chi-square value is influenced by the sample size, which indicates the fit of the data to the proposed model; therefore, its ratio to the degree of freedom gives more reliable results^53,54^. According to the structural validity of our test, all fit indices were acceptable except for the chi-square test results. The RMSEA value was 0.069, which is acceptable, while in a previous study, it was 0.03, which indicates good fit^33^. In this study, the TLI and CFI ranged between 0.952 and 0.980, indicating good fit, which is supported by the results of previous studies, where these indices also showed a good fit with values above 0.97^31,33^. The psychometric testing of the questionnaire demonstrated that the measure is psychometrically acceptable, reliable and valid for measuring turnover intention among Hungarian elderly care institution workers.

### Strengths and limitations

To date, there has been no Hungarian questionnaire suitable for assessing this problem, which was carried out with the involvement of 269 workers, which can be considered a relatively large number of participants. The participants were from all over Hungary, and were working in the field of elderly care. The limitations of the study include the nonnormal distribution of the data. The study included only Hungarian elderly care institution workers; therefore, the test results cannot be generalized to healthcare providers working in hospitals and clinics or workers in other areas of social work, e.g., homeless people or people with disability care institutions. Workers in elderly care and social work are mostly females; therefore, the study included mostly female participants, even though the study participants were recruited from all over the country. Future investigations, which are directed specifically to male workers in elderly care institutions, in addition to workers in health care institutions or other areas of social care, are recommended. We recommend also investigating the costs of turnover in elderly care and healthcare institutions in Hungary.

## Conclusions

The Hungarian version of the TIS-6 is a valid and reliable tool for assessing the turnover intentions of workers in the field of elderly care in Hungary. The questionnaire helps employees recognize and assess their turnover intention, which can provide useful information for management.

### Supplementary Information

Supplementary Table S1.

## Data Availability

The quantitative statistical data used to support the findings of this study are available from the corresponding author upon request.
