# Supplementary material for: Validation of the Hungarian version of the 6-item turnover intention scale among elderly care workers
Source: Sci Rep. 2024 Jul 6;14:15593. doi: 10.1038/s41598-024-66671-0 (PMC11227566; doi:10.1038/s41598-024-66671-0)
Supplement: Supplementary file 1 — Supplementary Table S1. [file 41598_2024_66671_MOESM1_ESM.pdf]

The adaptation of the 6 item Turnover Intention Scale among Hungarian elderly care workers: A validity and reliability study

The final Hungarian version of the shortened Turnover Intention Scale (TIS-6)

English version of the shortened Turnover Intention Scale (TIS-6)

Turnover Intention Scale [19]

The following section aims to ascertain the extent to which you intend to stay at the organization. Please read each question and indicate your response using the scale provided for each question:

During the past 9 months...

1	How often have you considered leaving your job?	**Never**	1-------2-------3-------4-------5	**Always**
2	How satisfying is your job in fulfilling your personal needs?	**Very satisfying**	1-------2-------3-------4-------5	**Totally dissatisfying**
3	How often are you frustrated when not given the opportunity at work to achieve your personal work-related goals?	**Never**	1-------2-------3-------4-------5	**Always**
4	How often do you dream about getting another job that will better suit your personal needs?	**Never**	1-------2-------3-------4-------5	**Always**
5	How likely are you to accept another job at the same compensation level should it be offered to you?	**Highly unlikely**	1-------2-------3-------4-------5	**Highly likely**
6	How often do you look forward to another day at work?	**Always**	1-------2-------3-------4-------5	**Never**

Hungarian version of the shortened Turnover Intention Scale (TIS-6)

Munkahelyváltási Szándék Skála

A skála célja, hogy megbizonyosodjon arról, milyen mértékben kíván a szervezetnél maradni.

Kérjük, olvasson el minden egyes kérdést, és a mellékelt skála alapján értékelje a választ.

Az elmúlt 9 hónap alatt…

1	Milyen gyakran gondolkozik munkahelyváltáson?	**soha**	1-------2-------3-------4-------5	**mindig**
2	Mennyire elégíti ki a munkája a személyes igényeit?	**nagyon kielégítő**	1-------2-------3-------4-------5	**teljességgel elégedetlen**
3	Milyen gyakran okoz csalódást, hogy nem kap lehetőséget a munkájában, személyes munkahelyi céljai megvalósításában?	**soha**	1-------2-------3-------4-------5	**mindig**
4	Milyen gyakran álmodozik arról, hogy a személyes igényeihez jobban illeszkedő munkát kap?	**soha**	1-------2-------3-------4-------5	**mindig**
5	Mennyire valószínű, hogy elfogadna egy másik felajánlott munkát, ugyanennyi fizetésért?	**nagyon valószínűtlen**	1-------2-------3-------4-------5	**nagyon valószínű**

6	Milyen gyakran várja örömmel a következő munkanapot?	**mindig**	1-------2-------3-------4-------5	**soha**
